# Unusually rapid β-cell failure in a patient newly diagnosed with type 2 diabetes presenting acutely with unprovoked severe hyperglycaemic hyperosmolar state: a case report

**DOI:** 10.4076/1757-1626-2-8880

**Published:** 2009-08-10

**Authors:** Joey Yeoh, Judy Chien-Chun Huang, Harriet Cheng, Kenneth Ross Muir

**Affiliations:** 1 Department of Endocrinology & Diabetes, Centre for Clinical Research & Effective Practice, Room 33, Support Building, Middlemore HospitalOtahuhu, South Auckland, Private Bag 93311New Zealand; 2 Department of Medicine, Middlemore HospitalOtahuhu, South Auckland, Private Bag 93311New Zealand

## Abstract

Pancreatic β-cell failure on a background of insulin resistance results in the inability to compensate for fasting hyperglycaemia and eventually produces type 2 diabetes mellitus. We describe an interesting case of a patient who presented acutely with unprovoked severe hyperglycaemic hyperosmolar state and was subsequently diagnosed with type 2 diabetes mellitus on a background of only impaired first phase insulin secretion 4 months prior. Glucagon stimulation test detected significant β-cell failure necessitating long term exogenous insulin therapy which is highly unusual by virtue of the rapid apparent deterioration.

## Introduction

Type 2 Diabetes Mellitus (T2DM) is undoubtedly the scourge of the developed world and a source of considerable socioeconomic burden [1]. It accounts for 90-95% of diabetes cases worldwide [2]. Although the exact aetiology of T2DM remains unknown, it involves a combination of insulin resistance and some degree of pre-existing β-cell secretory dysfunction conferring a state of relative rather than absolute insulin deficiency which progressively deteriorates with time irrespective of treatment [2-5]. Individuals at risk of developing overt T2DM, including the pre-diabetic states of impaired glucose tolerance (IGT) and impaired fasting glycaemia (IFG) already exhibit β-cell dysfunction and the weight of evidence suggests that this occurred long before the onset of pre-diabetes, when normal fasting glucose was still present [5,6].

Pancreatic β-cell compensation initially keeps glycaemia near normal despite underlying insulin resistance by increasing insulin secretion during the normoglycaemic and pre-diabetes stages [3-5]. The failure of β-cells to compensate at some point leads to development of overt T2DM [5,6]. This β-cell failure develops in a progressive fashion and continues after diagnosis, frequently resulting in secondary failure and exogenous insulin requirement [5,6]. It has not been reported <6 months from detection of impaired glucose metabolism (IGM) [7]. We report a case where the duration between onset of initial abnormality in glucose homeostasis and apparent β-cell failure necessitating long term exogenous insulin therapy is unusually rapid.

## Case presentation

A 73-year-old Caucasian male presented to the Emergency Department (ED) in June 2008 with a history of collapse without loss of consciousness while mobilizing to the toilet in the early hours of the morning. This occurred on the background of a 6 day history of general unwellness. He was conscious and orientated on arrival but was slightly drowsy. Other than clinical features of dehydration and mild central abdominal adiposity with a waist: hip ratio of 1.04, there were no other significant clinical signs. His random plasma glucose (RPG) on arrival was 59.1 mmol/L. His height was 1.76 meters (m) and weight was 90 kilograms (kg) making the body mass index (BMI) 29.0 kg/m^2^. The patient was previously fit and well without any known co-morbidities. He was on no regular medication. There was no family history of diabetes or other significant conditions. He was a non-smoker and consumed no alcohol. He works as a farm equipment evaluator. There was no history of specific weight loss over the preceding months. His general practitioner (GP) had performed a fasting plasma glucose test on him in February 2008 which showed a value of 6.4 mmol/L. This was repeated within 2 weeks and the repeated value was 6.1 mmol/L. Glycosylated haemoglobin (Hb_A1c_) was also done and was 6.3%. Subsequent 75 gram (g) oral glucose tolerance test (OGTT) produced a value of 15.3 mmol/L, 14.1 mmol/L and 7.6 mmol/L 30 minutes, 1-hour and 2-hours post glucose load respectively. He was managed with dietary modification and exercise.

His sodium (Na) was 150 mmol/L, potassium (K) 4.6 mmol/L, urea 26.1 mmol/L and creatinine (Cr) 237 micromol/L. Calculated osmolality was 394.4 mOsm/kg. Arterial blood gas sampling (ABG) showed a pH of 7.36, pCO2 4.9 kPa, pO2 11.0 kPa, bicarbonate 20 mmol/L and BE -1 mmol/L. Lactate was mildly elevated at 1.4 mmol/L. β-hydroxybutyrate (β-OHB) was slightly elevated at 3.67 mmol/L. Repeat HBA1c was 8.1%. Besides a small amount of ketones and glycosuria, urine testing revealed nil else of concern. Full septic screen was done; white cell count (WCC) and differential showed mildly raised WCC at 12.5 × 109 and neutrophils at 10.9 × 109 with the rest of differential count being normal, CRP was 4, urine microscopy, culture and sensitivity was normal and chest radiograph was normal. Thyroid function was normal. Lipid profile was also normal.

The patient was diagnosed with T2DM based on his presentation, age and absence of anti-Glutamic Acid Decarboxylase 65 (Anti-GAD 65), Insulinoma Antigen-2 (IA-2) and Islet Cell (ICA) antibodies. His acute presentation was consistent with hyperglycaemic hyperosmolar state (HHS) and acute renal failure secondary to dehydration. He was treated with intravenous insulin and intensively rehydrated with close attention to electrolyte balance. He improved considerably and renal function normalized. He was started on Metformin 500 mg twice daily (BID) increasing to 1 g BD and Penmix 30 subcutaneous insulin at a dose of 12 units BD increasing to 30 units BD. 5 days after the acute event, a 1 mg intravenous (IV) glucagon stimulation test was done at 9.00 am with blood samples for glucose and C-peptide taken at baseline, 5, 10 and 15 minutes.

Patient had been fasted for 15 hours overnight and medications were withheld until after the test. The glucose values were 6.3 mmol/L, 15.1 mmol/L, 16.3 mmol/L and 16.6 mmol/L at baseline, 5, 10 and 15 minutes respectively. Corresponding C-peptide values were 0.21 nmol/L, 0.42 nmol/L, 0.47 nmol/L and 0.51 nmol/L respectively. He was finally discharged after 7 days of hospital care with clinic follow up in 2 months. His average capillary blood glucose (CBG) was 6.7 mmol/L and range 6.5-7.7 mmol/L on discharge.

## Discussion

The OGTT results prior to admission provided surrogate information on first phase insulin secretion with the relative inability to clear glucose 30 minutes post load being suggestive of first phase insulin secretory defect characteristic of IGM [8]. At that stage, the patient could be classified as having mixed IFG/IGT but did not meet criteria for diagnosis of T2DM [2]. Subsequent admission with overt, apparently unprovoked HHS and full blown T2DM requiring insulin to achieve glycaemic control within 4 months of identification of IGM in a previously treatment naïve individual is highly unusual [6]. In addition, the IV glucagon stimulation test demonstrated the inability of β-cells to respond to hyperglycaemia which not only confirmed established T2DM, but also significant β-cell failure [3-5].

Homeostasis model assessment-insulin resistance (HOMA-IR), reciprocal HOMA-IR and quantitative insulin sensitivity check index (QUICKI) were not done to confirm insulin resistance or state of insulin sensitivity since these calculations require intact β-cell function (fasting insulin and glucose levels) [9]. Other complex tests and calculations for insulin sensitivity/insulin resistance or for assessment of β-cell function were not performed as they were deemed to be more appropriate in controlled research settings and beyond the ethical scope of a case report.

Pancreatic β-cell function is reported to be about 50%-60% of normal at the time of diagnosis of T2DM regardless of severity of insulin resistance [3,4,6]. The possible cause(s) of progressive β-cell dysfunction and failure are a matter of intense interest due to the obvious potential for therapy. The β-cell dysfunction and eventual failure involves β-cell secretory defects (manifesting as loss of first and then second phases of insulin secretion, reduced diurnal oscillations and impaired rapid pulsatile secretion) and reduction of β-cell mass [3-6]. Research has revealed several potential causes including glucotoxicity, lipotoxicity, proinflammatory cytokines secreted by adipose tissue and immune system and islet amyloid deposition [3-6]. Recent research even provided a possible cellular level common pathway by which β-cell failure can occur, involving the unfolded protein response (UPR) signalling pathways in the endoplasmic reticulum (ER) [10]. Regardless, it is fair to say that we do not yet know the exact cause(s) of β-cell dysfunction and failure. With regards to the possible factor(s) we do know about, there is no conclusive data about the actual duration needed to cause significant β-cell dysfunction and hence onset of T2DM, although the process is thought to occur over years rather than months [3-6]. In this patient, the rapid apparent deterioration of β-cell function cannot be adequately explained by lipotoxicity or glucotoxicity since there was no hyperlipidaemia or significantly raised plasma glucose previously. The possibility that acute glucotoxicity during the HHS affected the IV glucagon stimulation test result is unlikely since the test was done 5 days later, when the patient already had good glycaemic control. Even if the above mentioned causes existed in this patient, the rapid progression profile makes it unlikely that they were of any major importance.

## Conclusion

Although progression from IGM to T2DM is an expected course in the spectrum of the disease, rapid β-cell failure and development of insulin requiring T2DM within a few months of IGM is unusual. Therefore, this case suggests the pervasive likelihood of other, yet undetected factor(s), which may cause uncharacteristically rapid β-cell failure and rapid progression from normal glycaemia to IGM and overt insulin requiring T2DM.

## Abbreviations

ABG: arterial blood gas
Anti-GAD 65: anti-glutamic acid decarboxylase 65
BID: Bis in Die
BMI: body mass index
ER: endoplasmic reticulum
Hb_A1c_: glycosylated haemoglobin
HHS: hyperglycaemic hyperosmolar state
HOMA-IR: homeostasis model assessment - insulin resistance
IA-2: insulinoma antigen-2
ICA: islet cell antibody
IFG: impaired fasting glycaemia
IGM: impaired glucose metabolism
IGT: impaired glucose tolerance
OGTT: oral glucose tolerance test
QUICKI: quantitative insulin sensitivity check index
RPG: random plasma glucose
T2DM: Type 2 Diabetes Mellitus
UPR: unfolded protein response
WCC: white cell count
